# Insulin-like Growth Factor 2 (IGF-2) Potentiates BMP-9-Induced Osteogenic Differentiation and Bone Formation

**DOI:** 10.1002/jbmr.133

**Published:** 2010-05-17

**Authors:** Liang Chen, Wei Jiang, Jiayi Huang, Bai-Cheng He, Guo-Wei Zuo, Wenli Zhang, Qing Luo, Qiong Shi, Bing-Qiang Zhang, Eric R Wagner, Jinyong Luo, Min Tang, Christian Wietholt, Xiaoji Luo, Yang Bi, Yuxi Su, Bo Liu, Stephanie H Kim, Connie J He, Yawen Hu, Jikun Shen, Farbod Rastegar, Enyi Huang, Yanhong Gao, Jian-Li Gao, Jian-Zhong Zhou, Russell R Reid, Hue H Luu, Rex C Haydon, Tong-Chuan He, Zhong-Liang Deng

**Affiliations:** 1 Department of Orthopaedic Surgery, Second Affiliated Hospital, Chongqing Medical University Chongqing, People's Republic of China; 2 Molecular Oncology Laboratory, Department of Surgery, The University of Chicago Medical Center Chicago, IL, USA; 3 Key Laboratory of Diagnostic Medicine Designated by the Chinese Ministry of Education and the Affiliated Hospitals, Chongqing Medical University Chongqing, People's Republic of China; 4 Department of Orthopaedic Surgery, West China Hospital, Sichuan University Chengdu, Sichuan, People's Republic of China; 5 Department of Radiology, The University of Chicago Chicago, ILUSA; 6 School of Bioengineering, Chongqing University Chongqig, People's Republic of China; 7 Department of Geriatrics, Xinhua Hospital of Shanghai Jiatong University Shanghai, People's Republic of China

**Keywords:** BMP-9, bone formation, fracture healing, IGF-2, osteoblastic differentiation

## Abstract

Efficient osteogenic differentiation and bone formation from mesenchymal stem cells (MSCs) should have clinical applications in treating nonunion fracture healing. MSCs are adherent bone marrow stromal cells that can self-renew and differentiate into osteogenic, chondrogenic, adipogenic, and myogenic lineages. We have identified bone morphogenetic protein 9 (BMP-9) as one of the most osteogenic BMPs. Here we investigate the effect of insulin-like growth factor 2 (IGF-2) on BMP-9-induced bone formation. We have found that endogenous IGF-2 expression is low in MSCs. Expression of IGF-2 can potentiate BMP-9-induced early osteogenic marker alkaline phosphatase (ALP) activity and the expression of later markers. IGF-2 has been shown to augment BMP-9-induced ectopic bone formation in the stem cell implantation assay. In perinatal limb explant culture assay, IGF-2 enhances BMP-9-induced endochondral ossification, whereas IGF-2 itself can promote the expansion of the hypertropic chondrocyte zone of the cultured limb explants. Expression of the IGF antagonists IGFBP3 and IGFBP4 leads to inhibition of the IGF-2 effect on BMP-9-induced ALP activity and matrix mineralization. Mechanistically, IGF-2 is further shown to enhance the BMP-9-induced BMPR-Smad reporter activity and Smad1/5/8 nuclear translocation. PI3-kinase (PI3K) inhibitor LY294002 abolishes the IGF-2 potentiation effect on BMP-9-mediated osteogenic signaling and can directly inhibit BMP-9 activity. These results demonstrate that BMP-9 crosstalks with IGF-2 through PI3K/AKT signaling pathway during osteogenic differentiation of MSCs. Taken together, our findings suggest that a combination of BMP-9 and IGF-2 may be explored as an effective bone-regeneration agent to treat large segmental bony defects, nonunion fracture, and/or osteoporotic fracture. © 2010 American Society for Bone and Mineral Research.

## Introduction

Bone is one of a few organs that retain the potential for regeneration into adult life and is the only tissue that can undergo continual remodeling throughout life. Osteogenesis is a sequential cascade that recapitulates most, if not all, of the cellular events occurring during embryonic skeletal development.(1) During skeletogenesis, bone formation can occur through two different pathways, intramembranous ossification and endochondral ossification.(1) Bone regeneration following a fracture progresses through sequential phases similar to endochondral ossification, starting with chemotaxis and proliferation of mesenchymal stem cells. Efficacious bone regeneration would have an important impact on the clinical management of many bone and musculoskeletal disorders, such as with segmental bone loss, fracture nonunion, osteoporotic fracture, and failed spinal fusion. Mesenchymal stem cells (MSCs) hold great promise for tissue bioengineering and regenerative medicine. MSCs are adherent marrow stromal cells that can self-renew and differentiate into osteogenic, chondrogenic, adipogenic, and myogenic lineages.(2–4) Several signaling pathways have been implicated in regulating stem cell self-renewal and lineage commitment.(5–8)

Bone morphogenetic proteins (BMPs) play an important role in regulating cell proliferation and differentiation during development(8–11) and have been shown to play an important role in stem cell biology.(12,13) BMPs belong to the transforming growth factor β (TGF-β) superfamily and consist of at least 15 members in humans.(8–11,14) Genetic disruptions of BMPs have resulted in various skeletal and extraskeletal abnormalities during development.(14,15) BMPs fulfill their signaling activity by interacting with the heterodimeric complex of two transmembrane serine/threonine kinase receptors, BMPR type I and BMPR type II.(9) The activated receptor kinases phosphorylate the transcription factors Smads 1, 5, or 8, which, in turn, form a heterodimeric complex with Smad4 in the nucleus and activate the expression of target genes in concert with other coactivators.(9) On analyzing the 14 types of BMPs, we found that BMP-9 is one of the most potent BMPs in inducing osteogenic differentiation of MSCs both in vitro and in vivo.(11,16–22) We further demonstrated that BMP-9 regulates a distinct set of downstream targets that may play a role in regulating BMP-induced osteoblast differentiation of MSCs.(11,18–21)

BMP-9 (also known as *growth differentiation factor 2*, or *GDF-2*) was first identified in the developing mouse liver,(23) and its possible roles include inducing and maintaining the cholinergic phenotype of embryonic basal forebrain cholinergic neurons,(24) inhibiting hepatic glucose production and inducing the expression of key enzymes of lipid metabolism,(25) and stimulating murine hepcidin 1 expression.(26) Although the functional role of BMP-9 in the skeletal system remains to be fully understood, the potent osteogenic activity of BMP-9 suggests that it may be used as an efficacious bone-regeneration agent. It is conceivable that other growth factors may act synergistically or enhance BMP-9-induced bone formation.

Here we sought to investigate the effect of insulin-like growth factor 2 (IGF-2) on BMP-9-induced bone formation. As a member of the IGF signaling system, IGF-2 plays an important role in prenatal growth and development.(27) IGF-2 transduces its signaling through IGF receptors and activates the phosphatidylinositol-3-kinase (PI3K)/AKT pathway or the mitogen-activated protein kinase (MAPK) pathway.(28) *Igf2* null mice exhibit a 40% decrease in birth weight compared with their wild-type littermates, suggesting an important role of IGF-2 in development.(29,30) We have found that endogenous IGF-2 expression is relatively low in MSCs. Exogenous expression of IGF-2 can potentiate BMP-9-induced early osteogenic marker alkaline phosphatase (ALP) activity and the expression of later markers, such as osteocalcin (OC) and osteopontin (OPN), in MSCs. IGF-2 is shown to augment BMP-9-induced ectopic bone formation in stem cell implantation studies. Using perinatal limb explant culture, we have demonstrated that IGF-2 enhances BMP-9-induced endochondral ossification, whereas IGF-2 itself can promote expansion of the hypertropic chondrocyte zone of cultured limb explants. Exogenous expression of IGFBP3 and IGFBP4, but not IGFBP5, leads to inhibition of the IGF-2 effect on BMP-9-induced ALP activity and matrix mineralization in MSCs. Furthermore, IGF-2 is shown to enhance the BMP-9-induced BMPR-Smad reporter activity and the nuclear translocation of Smad1/5/8. While BMP9 stimulation does not significantly induce AKT phosphorylation, PI3K inhibitor LY294002 abolishes the IGF2 potentiation effect on BMP9-mediated osteogenic signaling, and can directly inhibit BMP9 activity, suggesting that BMP-9 may crosstalk with IGF-2 through the PI3K/AKT signaling pathway during osteogenic differentiation of MSCs.

## Materials and Methods

### Cell culture and chemicals

HEK293, C3H10T1/2, and C2C12 cells were obtained from ATCC (Manassas, VA, USA). Cell lines were maintained under conditions as described previouslyy.(16,18,22,31,32) Unless indicated otherwise, all chemicals were purchased from Sigma-Aldrich (St Louis, MO, USA) or Fisher Scientific (Pittsburgh, PA, USA).

### Recombinant adenoviruses expressing *RFP*, *GFP*, *BMP9*, *IGF2*, *IGFBP3*, *IGFBP4*, and *IGFBP5*

Recombinant adenoviruses were generated using AdEasy technology, as described previously.(16,17,33–35) The coding regions of human *BMP9*, *IGF2*, *IGFBP3*, *IGFBP4*, and *IGFBP5* were PCR amplified and cloned into an adenoviral shuttle vector and subsequently used to generate recombinant adenoviruses in HEK293 cells. The resulting adenoviruses were designated as AdBMP9, AdR-IGF2, AdR-IGFBP3, AdR-IGFBP4, and AdR-IGFBP5. AdBMP9 also expresses GFP, whereas AdR-IGF2, AdR-IGFBP3, AdR-IGFBP4, and AdR-IGFBP5 express RFP as a marker for monitoring infection efficiency. Analogous adenovirus expressing only monomeric RFP (AdRFP) or GFP (AdGFP) were used as controls.(19–22,33,35–38)

### Isolation of mouse embryo fibroblasts (MEFs)

MEFs were isolated from postcoitus day 13.5 mice, as described previously.(21,22) Each embryo was dissected into 10 mL of sterile PBS, voided of its internal organs, and sheared through an 18-gauge syringe in the presence of 1 mL of 0.25% trypsin and 1 mM EDTA. After 15 minutes of incubation with gentle shaking at 37°C, DMEM with 10% fetal calf serum (FCS) was added to inactivate trypsin. The cells were plated on 100-mm dishes and incubated for 24 hours at 37°C. Adherent cells were used as MEF cells. Aliquots were kept in a liquid nitrogen tank. All MEFs used in this study were within five passages.

### RNA isolation and semiquantitative RT-PCR analysis

Total RNA was isolated using TRIZOL Reagents (Invitrogen, Carlsbad, CA, USA). Total RNA was used to generate cDNA templates by RT reaction with hexamer and Superscript II RT (Invitrogen). The first-strand cDNA products were further diluted 5- to 10-fold and used as PCR templates. Semiquantitative RT-PCR was carried out as described previously.(21,22,31,34,38–41) PCR primers (Supplemental Table S1) were designed by using the Primer3 program (Free Software Foundation, Inc., Boston, MA, USA) to amplify the genes of interest (approximately 150 to 180 bp). A touchdown cycling program was as follows: 94°C for 2 minute for 1 cycle, 92°C for 20 seconds, 68°C for 30 seconds, and 72°C for 12 cycles with a decrease in 1°C per cycle and then at 92°C for 20 seconds, 57°C for 30 seconds, and 72°C for 20 seconds for 20 to 25 cycles depending on the abundance of a given gene. The specificity of PCR products was confirmed by resolving PCR products on 1.5% agarose gels. All samples were normalized by the expression level of *GAPDH*.

### Alkaline phosphatase (ALP) assay

ALP activity was assessed by a modified Great Escape SEAP Chemiluminescence Assay (BD Clontech, Mountain View, CA, USA) and/or histochemical staining assay (using a mixture of 0.1 mg/mL of napthol AS-MX phosphate and 0.6 mg/mL of Fast Blue BB salt), as described previously.(16,17,19–22,31,34,38) For the chemilluminescence assays, each assay condition was performed in triplicate, and the results were repeated in at least three independent experiments. ALP activity was normalized by total cellular protein concentrations among the samples.

### Transfection and luciferase reporter assay

Exponentially growing cells were seeded in 25-cm^2^ cell culture flasks and transfected with 2 µg per flask of BMP Smad-responsive luciferase reporter(42) p12xSBE-Luc using LipofectAmine (Invitrogen). At 16 hours after transfection, cells were replated to 24-well plates and infected with AdBMP9, AdR-IGF2, and/or AdRFP at 4 hours after replating. At 24 hours after infection, cells were lysed, and cell lysates were collected for luciferase assays using Promega's Luciferase Assay Kit. Each assay condition was performed in triplicate. The results were repeated in at least three independent experiments. Luciferase activity was normalized by total cellular protein concentrations among the samples. Reporter activity was expressed as mean ± SD.

### Matrix mineralization assay (alizarin red S staining)

C3H10T1/2 cells and MEFs were seeded in 24-well culture plates and infected with AdGFP, AdBMP9, and/or AdR-IGF2. Infected cells were cultured in the presence of ascorbic acid (50 µg/mL) and β-glycerophosphate (10 mM). At 14 days after infection, mineralized matrix nodules were stained for calcium precipitation by means of alizarin red S staining, as described previously.(16,17,19–22,31,34,38) Cells were fixed with 0.05% (v/v) glutaraldehyde at room temperature for 10 minutes. After being washed with distilled water, fixed cells were incubated with 0.4% alizarin red S (Sigma-Aldrich) for 5 minutes, followed by extensive washing with distilled water. The staining of calcium mineral deposits was recorded under bright-field microscopy.

### Immunohistochemical staining

Cultured cells were infected with adenoviruses. At the indicated time points, cells were fixed with 10% formalin and washed with PBS. The fixed cells were permeabilized with 1% NP-40 and blocked with 10% goat serum, followed by incubation with an antiosteocalcin, osteopontin, Smad1/5/8, or *p*-AKT1/2/3 (Ser-473) antibody (all from Santa Cruz Biotechnology, Santa Cruz, CA, USA) for 1 hour. After washing, cells were incubated with biotin-labeled secondary antibody for 30 minutes, followed by incubating cells with streptavidin–horseradish peroxidase (HRP) conjugate for 20 minutes at room temperature. The presence of the expected protein was visualized by 3,3′-Diaminobenzidine (DAB) staining and examined under a microscope. Stains without the primary antibody or with control IgG were used as negative controls.

### Subcutaneous stem cell implantation

C3H10T1/2 cells were infected with adenoviruses as indicated. At 16 hours after infection, cells were harvested and resuspended in PBS for subcutaneous injection (5 × 10^6^/injection) into the flanks of athymic nude (*nu*/*nu*) mice (five animals per group, 4- to 6-week old males, Harlan Sprague-Dawley, Indianapolis, IN, USA). At 5 weeks after implantation, animals were euthanized, and the implantation sites were retrieved for histologic evaluation and other stains.

### Fetal limb explant culture

The skinned forelimbs of mouse embryos (E18.5) were dissected under sterile conditions and incubated in DMEM (Invitrogen) containing 0.5% bovine serum albumin (BSA, Sigma), 50 µg/mL of ascorbic acid (Sigma), 1 mM β-glycerophosphate, and 100 µg/mL of penicillin-streptomycin (Mediatech, Manassas, VA, USA) solution at 37°C in humidified air with 5% CO_2_ for up to 14 days. The limb explants were directly infected by AdR-IGF2 and/or AdBMP9 one day after dissection. Then 100 mM calcein (Sigma) was added in the medium on the same day. The medium was changed in half volume on day 7. Cultured tissues were observed at different time points under the microscope to confirm the survival of tissue cells and the expression of fluorescence markers.

### Micro–computed tomographic (µCT) imaging analysis

All specimens were imaged using the µCT component of a GE Triumph (GE Healthcare, Piscataway, NJ, USA) trimodality preclinical imaging system. All image data analysis was performed using Amira 5.2 (Visage Imaging, Inc., San Diego, CA, USA), and 3D volumetric data were obtained.

### Histologic evaluation and trichrome staining

Retrieved and cultured tissues were fixed in 10% formalin (decalcified, if necessary) and embedded in paraffin. Serial sections of the embedded specimens were stained with hematoxylin and eosin. Paraffin-embedded sections were deparaffinized and then rehydrated in a graduated fashion. The deparaffinized samples were subjected to antigen retrieval and fixation. The sections were stained with hematoxylin and eosin (H&E) and Masson's trichrome.

### Quantitative analysis of image data using NIH ImageJ

The image analysis software ImageJ (http://rsbweb.nih.gov/ij/download.html) was used to analyze the regions of interest and/or intensity of the acquired image data. Random sampling of 10 to 20 fields were used routinely to determine mean ± SD values for statistical analysis.

### Statistical analysis

Microsoft Excel was used to calculate standard deviations (SDs) and statistically significant differences between samples using the two-tailed Student's *t* test. For all quantitative assays, each assay condition was performed in triplicate, and the results were repeated in at least three independent experiments. All data collected were subjected to statistical analysis. A *p*-value of less than .05 was defined as statistically significance.

## Results

### Endogenous IGF-2 expression is low in mesenchymal stem cells, and exogenous IGF-2 expression potentiates BMP-9-induced early osteogenic marker ALP activity

**Fig. 1:**
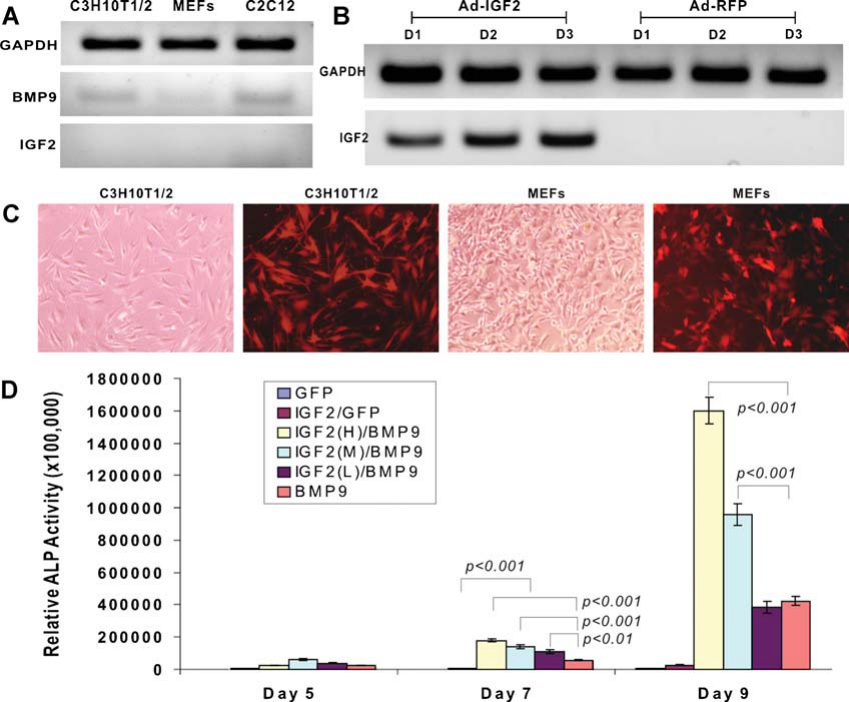
Potentiation of BMP-9-induced early osteogenic marker ALP activity in mesenchymal stem cells by IGF-2. (*A*) Endogenous expression of BMP-9 and IGF-2 in mesenchymal progenitor cells. Total RNA was isolated from C3H10T1/2 cells, MEFs, and C2C12 cells. Semiquantitative RT-PCR was performed using primers specific for mouse *GAPDH*, mouse *BMP9*, and mouse *IGF2*. The PCR products were resolved on 1% agarose gels and visualized under an ultraviolet lamp. (*B*) Exogenous expression of IGF-2 mediated by adenovirus-expressing human IGF-2. C3H10T1/2 cells were infected with AdR-IGF2 or Ad-RFP. Total RNA was isolated on days 1, 2, and 3 after infection and subjected to RT-PCR reactions. Exogenous expression of human IGF-2 was assessed by semiquantitative RT-PCR using primer pairs specific for human *IGF2*. The PCR products were resolved on 1% agarose gels and visualized under an ultraviolet lamp. (*C*) AdR-IGF2-mediated efficient transduction of MSCs. Subconfluent C3H10T1/2 and MEF cells were infected with recombinant adenovirus expressing human IGF-2 (AdR-IGF2) at a multiplicity of infection (MOI) of 10. The expression of marker gene monomeric *RFP* was detected at 36 hours after infection under both bright and fluorescence fields. (*D*) IGF-2 potentiates BMP-9-induced ALP activity in a dose-dependent fashion. C3H10T1/2 cells were coinfected with AdBMP9 and AdR-IGF2 at MOIs of 20 (high), 10 (medium), and 5 (low) dosages or GFP. ALP activity was measured at the indicated time points. Each assay condition was done in triplicate.

In order to determine the effect of IGF-2 on BMP-9-induced osteogenic differentiation and bone formation, we first examined the endogenous expression level of IGF-2 on mesenchymal progenitor cells. Using semiquantitative RT-PCR, we found that IGF-2 expression level was undetectable in C3H10T1/2 cells, mouse embryonic fibroblasts (MEFs), and C2C12 cells (Fig. 1*A*). However, under the same RT-PCR condition, the endogenous expression of BMP-9 was seemingly low but detectable, particularly in C3H10T1/2 and C2C12 cells. These results suggest that IGF-2 expression may be very low in MSCs.

To express exogenous IGF-2 effectively in MSCs, we constructed and generated a recombinant adenovirus-expressing human IGF-2 (ie, AdR-IGF2) using our previously developed AdEasy system.(33,35,43) As shown in Fig. 1*B*, IGF-2 expression was readily detected in AdR-IGF2 but not in Ad-RFP-infected C3H10T1/2 cells. Moreover, AdR-IGF2 was shown to effectively transduce C3H10T1/2 cells and MEFs (Fig. 1*C*).

We next tested the effect of exogenous IGF-2 on BMP-9-induced osteogenic differentiation of MSCs. Subconfluent C3H10T1/2 cells were coinfected with AdGFP, AdBMP9, and/or AdR-IGF2 (at high, medium, and low dosages). The activity of the well-established early osteogenic marker alkaline phosphatase (ALP)(16–20,22) was measured on days 5, 7, and 9 after infection. IGF-2 expression alone did not induce any detectable ALP activity over the GFP control (Fig. 1*D*). As expected, BMP-9 induced a significant increase in ALP activity, especially on day 9 (Fig. 1*D*). When BMP-9 expression was fixed, an increase in IGF-2 expression significantly induced an increase in ALP activity (Fig. 1*D*). Thus these results strongly indicate that IGF-2 can potentiate BMP-9-induced osteogenic differentiation in a dose-dependent fashion.

### Exogenous IGF-2 expression enhances BMP-9-induced late osteogenic marker expression and matrix mineralization

**Fig. 2:**
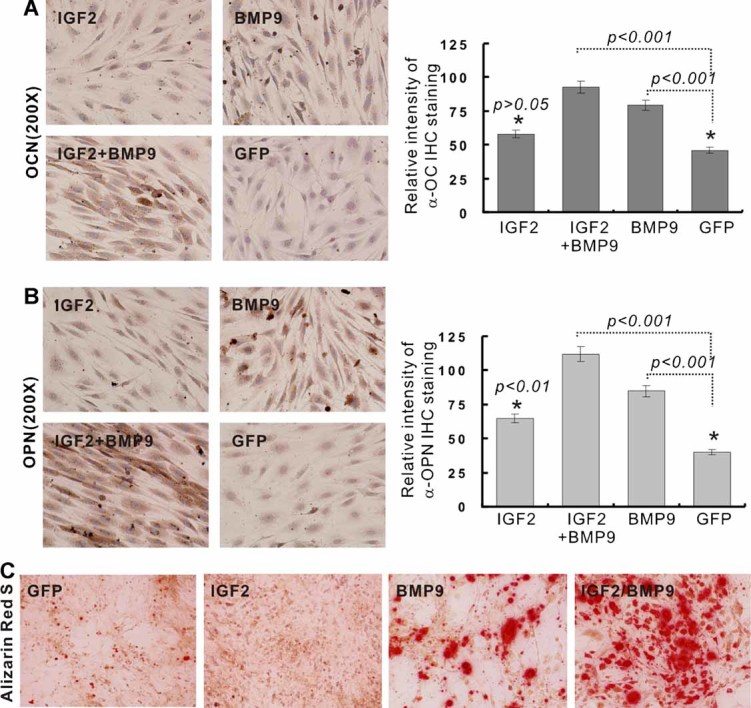
Augmentation of BMP-9-induced late osteogenic markers by IGF-2. (*A*) Immunohistochemical staining of osteocalcin. C3H10T1/2 cells were infected with adenoviruses as indicated. Expression of osteocalcin was assessed by immunohistochemical staining analysis at 10 days after infection using an antiosteocalcin antibody (Santa Cruz Biotechnology). (*B*) Immunohistochemical staining of osteopontin. C3H10T1/2 cells were infected with adenoviruses as indicated. Expression of osteopontin was assessed by immunohistochemical staining analysis at 10 days after infection using an antiosteopontin antibody (Santa Cruz Biotechnology). (*C*) Alizarin red S staining. C3H10T1/2 cells were infected with adenoviruses as indicated. Alizarin red S staining was conducted at 14 days after infection.

We further determined the effect of IGF-2 on the BMP-9-induced late stage of osteogenic differentiation. Both osteopontin and osteocalcin are well-established markers of late-stage bone formation.(16–20,22) We infected C3H10T1/2 cells with AdGFP, AdBMP9, and/or AdR-IGF2. The infected cells were fixed 10 days after infection and subjected to immunohistochemical staining using osteocalcin and osteopontin antibodies. Osteocalcin expression was detected in BMP-9- and BMP-9/IGF-2-transduced cells, whereas a higher than basal level staining was observed in IGF-2-stimulated cells (Fig. 2*A*). Osteopontin expression was detected in BMP-9/IGF-2-transduced cells and to a lesser extent in BMP-9- or IGF-2-stimulated cells (Fig. 2*B*). We also examined the effect of IGF-2 on BMP-9-induced matrix mineralization in MSCs. C3H10T1/2 cells were infected with AdGFP, AdBMP9, and/or AdR-IGF2. At 14 days after infection, the infected cells were subjected to alizarin red S staining. Numerous mineralization nodules were readily detected in BMP-9/IGF-2-stimulated cells, whereas a significant number of nodules also were found in BMP-9-transduced 3H10T1/2 cells (Fig. 2*C*). These findings strongly suggest that while IGF-2 itself has very limited ability to induce the late stage of bone formation, a combination of BMP-9 and IGF-2 significantly promotes the BMP-9-induced late stage of osteogenic differentiation in vitro.

### IGF-2 expression augments BMP-9-induced ectopic bone formation and matrix mineralization in MSC implantation in vivo

**Fig. 3:**
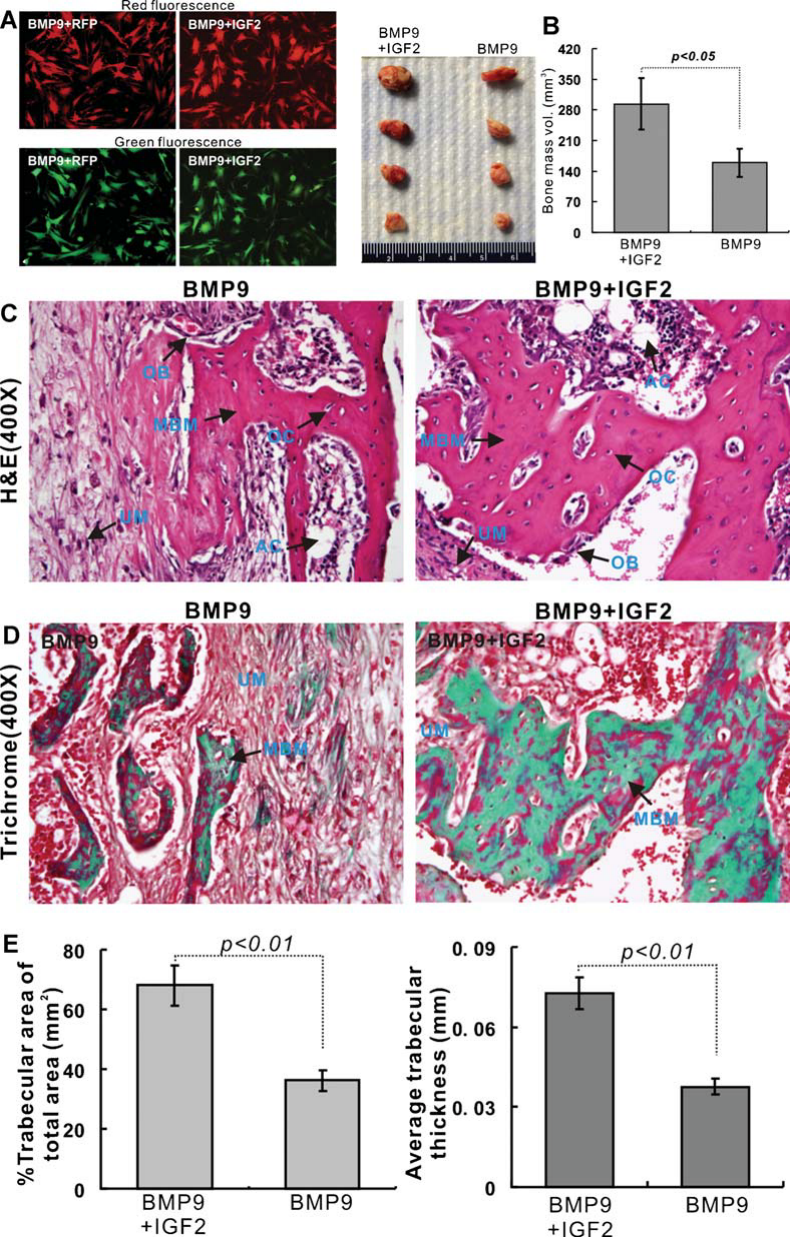
IGF-2 enhances BMP-9-induced ectopic bone formation. (*A*) Efficient transduction of MSCs via coinfection. Subconfluent C3H10T1/2 cells were coinfected with BMP-9 (GFP) and IGF-2 (RFP) adenoviruses or other combinations. Fluorescence images were recorded at 24 hours after infection. Representative images are shown. (*B*) Macrographic images of ectopic bone mass. BMP-9- or BMP-9/IGF-2-expressing MSCs were implanted subcutaneously. Ectopic osseous masses were retrieved at 5 weeks. Representative images from injection pairs are shown. Quantitative analysis of the bony masses also was done using µCT imaging analysis. (*C*) Histologic evaluation of the retrieved bone masses. Retrieved bone masses from BMP-9- or BMP-9/IGF-2-treated groups were fixed and decalcified. The paraffin-embedded sections were subjected to hematoxylin and eosine (H&E) staining. Representative images are shown. Magnification, ×300. (*D*) Masson's trichrome staining of the bone masses. The tissue sections prepared in panel *C* were subjected to Masson's trichrome staining. Representative images are shown. Magnification, ×300. (*E*) Quantitative analysis of percent trabecular area over total area and average trabecular thickness was done by using ImageJ. At least 10 samples (with ×40 magnification) from each group were randomly selected and analyzed using ImageJ software. AC = adipocyte; MBM = mineralized bone matrix; OB = osteoblast; OC = osteocyte; UM = undifferentiated MSCs.

Using our previously established stem cell implantation assay, we tested the effect of IGF-2 on BMP-9-induced ectopic bone formation in vivo. C3H10T1/2 cells were shown to be effectively coinfected with AdRFP, AdBMP9, and/or AdR-IGF2 (Fig. 3*A*). The infected cells were collected and injected subcutaneously into athymic nude mice. At week 5, the animals were euthanized, and the bony masses were retrieved (Fig. 3*B*). Consistent with our earlier reports, C3H10T1/2 cells transduced with either RFP or IGF-2 alone did not form any detectable masses (data not shown). The BMP-9-transduced cells formed bony masses, which were noticeably smaller than those formed by the cells transduced by both BMP-9 and IGF-2 (Fig. 3*B*). On histologic examination, bone masses formed in the BMP-9-transduced cell group showed some mature bone matrices and trabeculae with the presence of a significant number of undifferentiated mesenchymal progenitor cells (Figs. 3*C*, *E*). On the other hand, cells stimulated by both BMP-9 and IGF-2 formed more mature bone matrices and thicker trabeculae, with minimal or residual undifferentiated mesenchymal progenitor cells (Fig. 3*C*). Masson's trichrome staining confirmed that IGF-2 significantly augmented BMP-9-induced matrix mineralization (Fig. 3*D, E*). The preceding in vivo results further confirmed the in vitro findings presented in Fig. 1*D* and Fig. 2, suggesting that IGF-2 may be able to potentiate osteogenic differentiation of MSCs induced by BMP-9.

### IGF-2 promotes expansion of the hypertrophic chondrocyte zone and enhances BMP-9-induced endochondral ossification in fetal limb explant culture

**Fig. 4:**
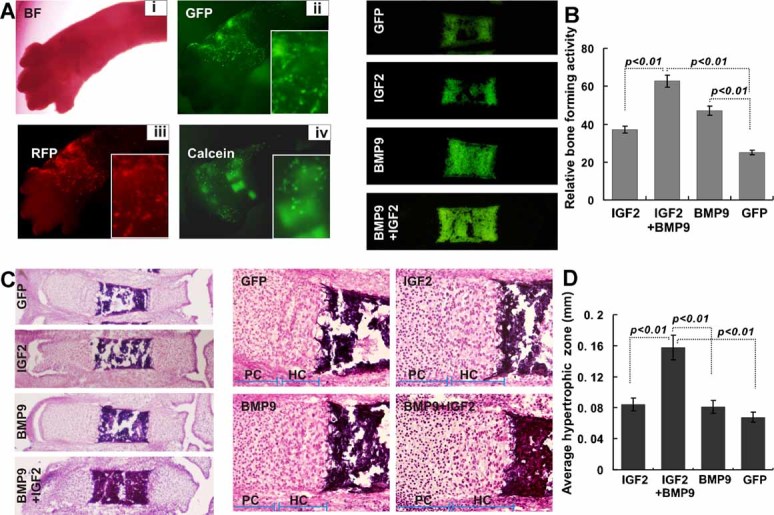
IGF-2 promotes expansion of the hypertrophic chondrocyte zone and enhances BMP-9-induced endochondral ossification in organ culture. (*A*) Harvest, transduction, and labeling of mouse E18.5 forelimbs (*n* = 8 each group). The E18.5 forelimbs were dissected, and the skin was removed with the soft tissues attached. The recombinant adenovirus (5 × 10^10^ pfu in 1 mL of medium) expressing GFP or RFP was added to culture medium. Bright-field (i), GFP fluorescence (ii), and RFP fluorescence (iii) images were taken at 36 hours after infection. The mineralization-labeling fluorescence dye calcein (0.5 µg/mL) was added to the medium, and green fluorescence signal was recorded at 24 hours after labeling (iv). (*B*) New bone formation of the cultured forelimbs stimulated with BMP-9 and/or IGF-2 at week 2. The dissected forelimb culture was infected with AdBMP9, AdGFP, AdR-IGF2, or AdBMP9/AdR-IGF2 for 2 weeks. Calcein (0.5 µg/mL) was added to the medium at 24 hours prior to harvest. The tissues were subjected to frozen section. The green fluorescence (calcein staining) signal from the fourth metacarpal of the cultured limbs is shown. Quantitative analysis of bone-forming activity (based on calcein incorporation) also was conducted by using ImageJ. (*C*, *D*) Histologic evaluation. The preceding frozen sections were subjected to H&E staining and recorded under bright field with ×40 magnification (*C*) and ×400 magnification (*D*). The average length of the hypertrophic zones also was determined by using ImageJ. HC = hypertrophic chondrocyte zone; PC = proliferating chondrocyte zone (partial).

IGF-2 signaling plays an important role in development.(28) We examined the effect of IGF-2 on skeletal development in fetal limb explant culture in the presence or absence of BMP-9. Perinatal E18.5 mouse embryos were harvested. The skinned fetal limbs were isolated and cultured in organ culture medium (Fig. 4*A*, i). The soft tissues associated with the cultured limb expants were infected with adenovirus expressing GFP (Fig. 4*A*, ii) or RFP (Fig. 4*A*, iii). The new bone formation was traced by the green fluorescence dye calcein (Fig. 4*A*, iv). When the limb explants were stimulated with GFP, IGF-2, and/or BMP-9 for 2 weeks, significant new bone formation (in the fourth metacarpal) was found in the limb explants infected with AdBMP9 and AdBMP9/AdR-IGF2, whereas the BMP-9/IGF-2-stimulated limb explants had the highest level of new bone formation, as judged by the calcein uptake (Fig. 4*B*). Histologic examination confirmed that increased endochondral ossification in BMP-9 and BMP-9/IGF-2 stimulated explants (Fig. 4*C*). At a higher magnification, either BMP-9 or IGF-2 stimulation significantly expanded the hypertrophic chondrocyte zone compared with that of the GFP control (Fig. 4*D*). These organ culture studies strongly suggest that IGF-2 may promote expansion of the hypertrophic chondrocyte zone and enhance BMP-9-induced endochondral ossification.

### IGFBP3 and IGFBP4 antagonize the effect of IGF-2 on BMP-9-induced osteogenic differentiation of MSCs

**Fig. 5:**
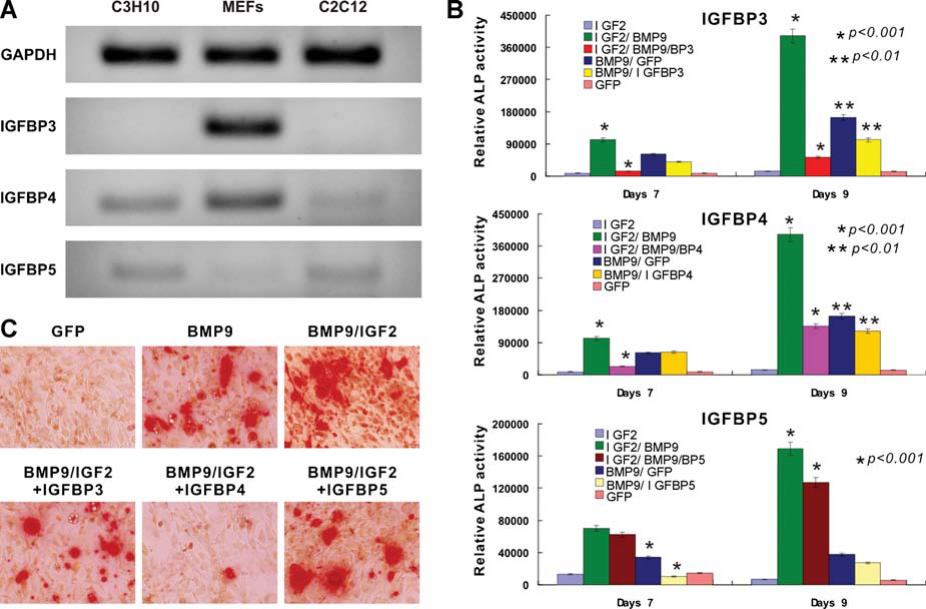
Inhibition of IGF-2-enhanced BMP-9-induced osteogenic differentiation by IGFBP3 and IGFBP4. (*A*) Endogenous expression of IGFBPs in MSCs. Total RNA was isolated from C3H10T1/2 cells, MEFs, and C2C12 cells and subjected to semiquantitative RT-PCR analysis using primers specific for mouse *GAPDH*, *IGFBP3*, *IGFBP4*, and *IGFBP5*. PCR products were resolved on 1.2% agarose gels. Representative data are shown. (*B*) IGFBP3 and IGFBP4 antagonize IGF-2-enhanced ALP activity induced by BMP-9. Subconfluent C3H10T1/2 cells were coinfected with adenoviruses expressing *IGF2*, *BMP9*, IGFBPs, and/or GFP. ALP activity was assessed on days 7 and 9. Each assay condition was done in triplicate. (*C*) IGFBP3 and IGFBP4 antagonize IGF-2-enhanced matrix mineralization induced by BMP-9. Subconfluent C3H10T1/2 cells were coinfected with adenoviruses expressing *IGF2*, *BMP9*, IGFBPs, and/or GFP in the presence of mineralization medium. Alizarin red S staining was performed on day 14. Representative data are shown.

IGF-2 signaling can be negatively regulated by IGFBPs. We analyzed the endogenous expression level of three IGFBPs. We found that IGFBP3 was highly expressed in MEFs but undetectable in both C3H10T1/2 and C2C12 cells (Fig. 5*A*). IGFBP4 was highly expressed in MEFs, whereas it was barely detectable in C3H10T1/2 and C2C12 cells (Fig. 5*A*). IGFBP5 expression was low or detectable in all three lines examined (Fig. 5*A*). These results indicate that the expression pattern of the three IGFBPs is different in the two progenitor lines C3H10T1/2 and C2C12 from that in the pool population of MEFs.

We next analyzed whether IGFBPs would antagonize IGF-2's effect on BMP-9-induced osteogenic differentiation. C3H10T1/2 cells were infected with AdGFP, AdR-IGF2, and/or AdBMP9 in the presence or absence of IGFBPs. At 7 or 9 days after infection, cells were collected and subjected to ALP activity assay. Both IGFBP3 and IGFBP4 were shown to inhibit IGF-2-enhanced BMP-9 osteogenic activity, whereas IGFBP5 exerted a minimal or insignificant inhibitory effect on IGF-2/BMP-9-induced osteogenic differentiation of C3H10T1/2 cells (Fig. 5*B*). A similar result also was found in alizarin red S staining assay because IGFBP3 and IGFBP4 were shown to inhibit BMP-9/IGF-2-induced matrix mineralization (Fig. 5*C*). In both assays, IGFBP4 was seemingly the strongest inhibitor of IGF-2's effect on BMP-9 osteogenic signaling. These results suggest that among the IGFBPs, IGFBP3 and IGFBP4 may be able to inhibit IGF-2-enhanced BMP-9-induced osteogenic differentiation.

### IGF-2 acts synergistically with BMP-9 in activating Smad signaling activity

**Fig. 6:**
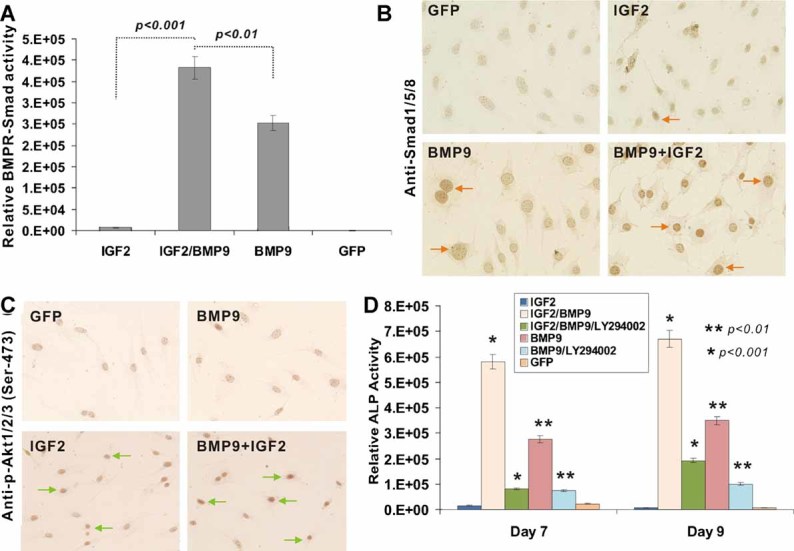
IGF-2-activated PI3K/AKT pathway crosstalks with BMP-9-mediated osteogenic signaling. (*A*) Effect of IGF-2- and BMP-9-induced BMPR-Smad reporter activity. Subconfluent C3H10T1/2 cells were seeded in 25-cm^2^ flasks and transfected with 2 µg per flask of the BMPR-Samd-responsive luciferase reporter p12xSBE-Luc using LipofectAmine (Invitrogen). At 16 hours after transfection, cells were replated to 24-well plates and were infected with AdGFP, AdBMP9, and/or AdR-IGF2 at 4 hours after replating. At 24 to 48 hours after infection, cells were lysed, and cell lysates were collected for luciferase assays using Promega's Luciferase Assay Kit. Each assay condition was performed in triplicate. (*B*) IGF-2 promotes BMP-9-induced nuclear localization of BMPR-Smads. Subconfluent C3H10T1/2 cells were infected with GFP, IGF2, and/or BMP9 for 16 hours. Cells were fixed and subjected to immunohistochemical staining using an anti-Smad1/5/8 antibody. Positive nuclear staining is indicated by arrows. Representative data are shown. (*C*) BMP-9 promotes IGF-2-induced phoshporylation of AKT. Subconfluent C3H10T1/2 cells were infected with AdGFP, AdR-IGF2, and/or AdBMP9 for 16 hours. Cells were fixed and subjected to immunohistochemical staining using an anti-*p*-AKT1/2/3 (Ser-473) antibody. Positive nuclear staining is indicated by arrows. Representative data are shown. (*D*) Inhibition of IGF-2-potentiated BMP-9-induced ALP activity by PI3K inhibitor. Subconfluent C3H10T1/2 cells were infected with AdGFP, AdR-IGF2, and/or AdBMP9. The PI3K inhibitor LY294002 (20 µM; BIOMOL, Plymouth Meeting, PA, USA) was added to AdR-IGF2/AdBMP9-transduced cells. At the indicated time points, ALP activity was determined. Each assay condition was done in triplicate.

To investigate the mechanism behind IGF2's effect on BMP-9-induced osteogenic signaling, we examined whether IGF-2 affected BMP-9 function at the Smad signaling level. We transfected C3H10T1/2 cells with a previously reported BMPR-Smad luciferase reporter, p12xSBE-Luc(42), and infected with GFP, IGF-2, and/or BMP-9 adenoviruses. As expected, BMP-9 was shown to significantly upregulate the BMPR-Smad reporter activity, whereas IGF-2 alone did not significantly activate BMPR-Smad reporter activity (Fig. 6*A*). However, IGF-2 was shown to effectively enhance BMP-9-induced BMPR-Smad reporter activity (Fig. 6*A*). Consistent with the results from R-Smad reporter assay, BMP-9 was shown to induce nuclear translocation of Smad1/5/8, which was further augmented by IGF-2 (Fig. 6*B*). IGF-2 alone failed to induce significant nuclear translocation of Smad1/5/8 (Fig. 6*B*). These results strongly suggest that IGF-2 may exhibit its stimulatory effect on BMP-9 signaling by modulating BMPR-Smad transcriptional activity.

### BMP-9 crosstalks with IGF-2 through the PI3K/AKT signaling pathway in MSCs

IGF-2 transduces its signaling through activation of the PI3K/AKT pathway. We sought to test whether BMP-9 crosstalks with IGF-2 through the PI3K/AKT pathway. When C3H10T1/2 cells were stimulated with GFP, IGF-2, and/or BMP-9 and subjected to immunohistochemical staining, the phosphorylated form of AKT1/2/3 (Ser-473) was detected in IGF-2-stimulated cells but not apparently in the control or BMP-9-stimulated cells (Fig. 6*C*). However, the phosphorylated AKT1/2/3 was most pronounced in the cells stimulated by both IGF-2 and BMP-9 (Fig. 6*C*), suggesting that there may be crosstalk between the BMP-9 and IGF-2 pathways.

Since AKT is regulated by PI3K, we further tested whether IGF-2-activated PI3K activity plays any role in BMP-9 signaling. Using a well-established PI3K inhibitor, LY294002, we infected C3H10T1/2 cells with GFP, BMP-9, and/or IGF-2 and treated them with LY294002 for 7 and 9 days. We found that the PI3K inhibitor LY294002 effectively inhibited the ALP activity in the cells stimulated by BMP-9 and IGF-2 (Fig. 6*D*). Interestingly, LY294002 also was shown to inhibit BMP-9-induced ALP activity (Fig. 6*D*, and data not shown), suggesting that PI3K may cross-regulate BMP-9-mediated osteogenic signaling.

## Discussion

In this study, we analyzed the effect of IGF-2 on BMP-9-induced bone formation. Through a comprehensive analysis of the 14 types of BMPs, we previously found that BMP-9 is one of the most osteogenic BMPs both in vitro and in vivo.(11,16–22) Our findings strongly suggest that BMP-9 may be used as an efficacious bone-regeneration agent. Nonetheless, it is conceivable that more efficacious bone regeneration may be achieved if BMP-9 is administered with other growth factors, such as IGF-2. Here we have found that endogenous IGF-2 expression is relatively low in MSCs. Exogenous expression of IGF-2 can potentiate BMP-9-induced activity of the early osteogenic marker ALP and the expression of later markers such as OC and OPN in MSCs. IGF-2 is shown to augment BMP-9-induced ectopic bone formation in our stem cell implantation assay. We have further demonstrated that IGF-2 enhances BMP-9-induced endochondral ossification, whereas IGF-2 itself can promote expansion of the hypertropic chondrocyte zone of the cultured limb explants. IGFBP3 and IGFBP4 can inhibit the IGF-2 effect on BMP-9-induced ALP activity and matrix mineralization in MSCs. IGF-2 is shown to enhance the BMP-9-induced BMPR-Smad reporter activity and the nuclear translocation of Smad1/5/8. PI3K inhibitor LY294002 abolishes the IGF-2 potentiation effect on BMP-9-mediated osteogenic signaling and can directly inhibit BMP-9 activity, suggesting that BMP-9 may crosstalk with IGF-2 through the PI3K/AKT signaling pathway during osteogenic differentiation of MSCs.

The IGF system consists of three ligands (IGF-1, IGF-2, and insulin), four cell-membrane receptors [IGF receptor type 1 (IGF-1R), insulin receptor isoform A (IR-A), hybrid receptors, and IGF receptor type 2 (IGF-2R)], and six IGF-binding proteins (IGFBP1 through -6).(27) It has been reported that TGF-β regulates IGFBP4 proteolysis in osteoblasts, which depends on IGF-2, suggesting that local TGF-β and IGF-2 in the bone microenvironment coordinately amplify IGF-1 bioavailability through controlled IGFBP4 proteolysis to promote bone formation.(44) This is supported by an earlier study in which systemic administration of IGFBP4 increased bone-formation parameters in mice by increasing IGF bioavailability in the circulation via an IGFBP-4 protease–dependent mechanism.(45) However, paracrine overexpression of IGFBP4 in osteoblasts of transgenic mice decreases bone turnover and causes global growth retardation.(46) As a member of the IGF signaling system, IGF-2 plays an important role in prenatal growth and postnatal development. IGF-2 transduces its signaling through IGF receptors and activates the PI3K/AKT pathway or the MAPK pathway.(27) Although viable and fertile, *Igf2* null mice exhibit a 40% decrease in birth weight compared with their wild-type littermates, suggesting an important role of IGF-2 in development.

The *IGF2* gene is imprinted and expressed only from the paternal allele in the placenta and fetal tissues, excluding the brain.(47) *IGF2* expression becomes biallelic in tissues in humans, but not rodents, after birth. Imprinting of the *IGF2* gene is controlled in part by the *H19DMR* (differentially methylated region) gene, which plays an important role in parent-of-origin-specific silencing of neighboring *H19* and *IGF2*. There are ontogenetic shifts in *IGF2* imprinting and *IGF* promoter usage that may influence IGF bioavailability in placental and fetal tissues at critical stages of development.(48,49) Loss of imprinting (LOI) of *IGF2* is strongly associated with cell proliferation and in many cases with human malignancies, including some sarcomas. The excess of IGF-2 in mice causes somatic overgrowth, visceromegaly, placentomegaly, omphalocele, and cardiac and adrenal defects, which are also features of the Beckwith-Wiedemann syndrome, a genetically complex human disorder associated with chromosomal abnormalities in the 11p15.5 region where the *IGF2* gene resides.(50) Mouse chimeras made with androgenetic (two paternal genomes) ova or embryonic stem cells frequently die at the perinatal stage and exhibit a range of defects, with the most noticeable being a pronounced overgrowth of rib cartilage, whole-body overgrowth, and perinatal death.(51) It has been reported that hypoxic stress enhanced osteoclast differentiation via increasing IGF-2 production by nonosteoclastic cells. The upregulation of IGF-2 derived from nonosteoclastic cells may be a crucial factor for osteoclast differentiation.(52) Furthermore, it has been reported that the local production of IGF-2 may modulate both osteoblast-osteoclast interactions and osteoclast formation and may play an important role in bone remodeling.(52) It has been reported recently that IGF-1 and BMP-2 can act synergistically in promoting chondrogenesis.(53,54) However, the molecular mechanisms of the IGF-2 stimulation that enhance osteoclast formation remain unknown.

IGF bioavailability is tightly controlled by IGFBPs, which are a family of six homologous multifunctional high-affinity proteins in the circulation. IGFBPs are mostly inhibitory to IGF actions but also have IGF-independent actions.(28) In the circulation, IGF-2 is bound mainly to IGFBP3, which is the most abundant IGFBP in serum. At the cellular level, the interaction of IGFs with their receptors is influenced by the presence of IGFBPs. Depending on the cellular context and the IGFBP present, the actions of IGFs can be either enhanced or inhibited. IGF-binding capacity of IGFBPs is regulated by the presence of a wide range of proteolytic enzymes. Increasing evidence suggests that IGFBPs also have IGF-independent functions. For example, IGFBP3 itself has a proapoptotic function, whereas IGFBP5 has a significant influence on bone mineral density (BMD) acquisition and maintenance in a gender- and age-dependent fashion, which may have implications for the gender-biased progression of osteoporosis.(55) Our results have demonstrated that IGFBP3 and IGFBP4, but not IGFBP5, can antagonize IGF-2 action on BMP-9-induced osteogenic differentiation. It was reported that IGFBP5 inhibits osteoblast differentiation and skeletal growth by blocking IGF actions.(56) Interestingly, an early report indicated that IGFBP3 potentiation of IGF action can be mediated through the PI3K pathway and is associated with alteration in AKT sensitivity.(57)

As one of the major pathways involved in IGF signaling, PI3K/AKT signaling is implicated in mediating a broad range of cellular responses, although its role in osteogenesis remains to be fully understood. In osteoclasts, PI3K functions as a critical downstream effecter of c-fms [the receptor for colony-stimulating factor 1 (CSF-1)], αvβ3 integrin, and RANK [receptor activator of nuclear factor κB (NF-κB)], affecting osteoclast survival and activity. PI3K/AKT signal relay may cooperate with Smad in BMP-2-induced CSF-1 expression and osteoclast differentiation.(58) Recent evidence suggests a role for PI3K/AKT in osteoblast differentiation and survival. It was reported that BMP-2-induced PI3K and AKT kinase are required in osteoblast differentiation and BMP-2/Smad-dependent gene transcription.(59) Runx2 induces osteoblast and chondrocyte differentiation and enhances their migration by coupling with PI3K/AKT signaling,(60) suggesting that Runx2 and PI3K/AKT signaling are mutually dependent on each other in the regulation of osteoblast and chondrocyte differentiation. It has been reported recently that an intact IGF-induced PI3K/AKT signaling cascade is essential for BMP-2-activated osteoblast differentiation and maturation, bone development, and growth.(61–65) Conversely, mice deficient in a negative regulator of PI3K signaling, the tumor suppressor *PTEN* in osteochondroprogenitor cells, exhibited epiphyseal growth plate abnormalities and skeletal overgrowth(66) and demonstrated a dramatic and progressively increasing BMD throughout life.(67) Taken together, these findings are consistent with our current results with the effect of IGF-2 on BMP-9-mediated osteogenic signaling, suggesting that manipulations of BMP and IGF pathways could facilitate bone remodeling and fracture repair.
